# The Evaluation of Thermal Stability, Electric Conductivity and Carbide Morphology of Austenitic Ductile Iron Castings

**DOI:** 10.3390/ma18204734

**Published:** 2025-10-15

**Authors:** Magdalena Bork, Marcin Górny, Łukasz Gondek, Jerzy Morgiel, Krzysztof Morgiel

**Affiliations:** 1 Faculty of Foundry Engineering, AGH University of Krakow, Mickiewicza Av. 30, 30-059 Krakow, Poland; mbork@agh.edu.pl (M.B.); k.morgiel23@gmail.com (K.M.); 2 Department of Solid State Physics, Faculty of Physics and Applied Computer Science, AGH University of Krakow, Mickiewicza Av. 30, 30-059 Krakow, Poland; lgondek@agh.edu.pl; 3 Institute of Metallurgy and Materials Science, Polish Academy of Sciences, 25 Reymonta St., 30-059 Krakow, Poland; j.morgiel@imim.pl

**Keywords:** Ni-resist ductile iron, thermal stability, electric conductivity, TEM, M_7_C_3_

## Abstract

The Ni-Resist ductile iron, with a nickel content ranging from 18% to 36%, is a material designed for service under extreme conditions. One of the main objectives of this study was to determine the minimum nickel content required to stabilize the austenitic structure at cryogenic temperatures. Additional aims included investigating structural features related to the solidification of austenite dendrites, graphite nodules, and eutectic carbides. Moreover, the electrical conductivity, which is critical for certain applications of Ni-Resist ductile irons, was also examined. To this end, castings with varying nickel content (21%, 25%, 28%, and 35%) and with or without chromium additions were prepared. Microstructural characterization was performed using optical, scanning, and transmission electron microscopy, X-ray diffraction (XRD), and electrical conductivity measurements. The results showed that a highly branched dendritic microstructure predominates, with graphite nodules located in interdendritic regions and along austenite grain boundaries. In chromium-alloyed ductile irons, the austenitic matrix contains Cr = 1.7 ± 0.3 wt.% in the vicinity of M_7_C_3_-type eutectic carbides. Furthermore, thermal stability analysis indicated that a minimum nickel content of 25 wt.% is sufficient to ensure structural stability at cryogenic temperatures down to 25 K. Finally, complementing the above-mentioned investigations, the electrical conductivity characteristics of the studied high-nickel austenitic cast irons were determined.

## 1. Introduction

Constant needs from industry for high performance materials led to choosing austenitic ductile iron over steel for various demanding applications, due to its outstanding combination of crucial properties, such as corrosion resistance, wear resistance and wide temperature stability [1,2,3,4,5,6].

Austenitic ductile irons, known as Ni-Resist ductile irons, are characterized by nickel addition from 18% up to 36%, and treatment with magnesium to create an austenitic metallic matrix and spheroidal graphite in the range from several dozen to several thousand graphite nodules per square millimeter [1]. The nodular graphite morphology, combined with the austenitic metallic matrix, results in numerous benefits, such as improved mechanical properties, high ductility, exceptional impact performance, and a wide range of operating temperatures—also under cryogenic conditions [6,7,8]. When carbide-promoting elements like Cr are added, carbides are also present in the microstructure. The austenitic matrix, which does not undergo transformation from austenite (γ) to ferrite (α) [8,9], can be retained due to a high nickel content (>18%). Nickel dissolves in liquid cast iron as well as in its solid solutions without limitations. It promotes graphitization during eutectic solidification, which reduces the tendency to form carbides (chill tendency). The solute partition coefficient of nickel is close to one, hence it shows little tendency to inverse microsegregation and also has little effect on the growth restriction factor, which reflects the solute rejection at the solid–liquid interface. Its high content is responsible for the stable austenitic metallic matrix. As a result, Ni-Resist ductile irons are capable of operating over a wide temperature range, up to 1050 °C [6].

Among the remarkable properties of Ni-Resist ductile irons are their excellent resistance to corrosion, wear, galling, erosion, and high temperatures, which are even higher for grades with chromium additions up to 5 wt.%, albeit at the expense of plastic and dynamic properties. This results from the fact that carbides formed as a consequence of the presence of chromium usually create a network along grain boundaries and in interdendritic regions. Heat treatment involving partial spheroidization of carbides may be carried out to improve service properties.

Thus, thanks to their superior properties, high-quality austenitic ductile iron castings, including thin-walled, are widely used in various applications, such as chemical processing (compressors, cryogenic equipment), the electrical power industry (non-magnetic housings, resistance grids), internal combustion engines (exhaust components such as manifolds and valve guides), the marine industry (pumps and pump components) and others [6].

The literature provides limited data [6,10,11,12,13] on the role of chemical composition (in particular nickel and chromium) on the structural stability and electrical conductivity, which indicates that Ni-Resist irons, despite their widespread industrial application, still require in-depth investigations.

Filling this gap, the present study focuses on structure and thermal stability analysis of high quality Ni-Resist castings with varying nickel content (21%, 25%, 28%, and 35%) and with or without chromium additions, produced according to the EN-GJSA-XNi22 and XNiSiCr35-5-2 Standards using optical, scanning, and transmission electron microscopy, and X-ray diffraction (XRD). Finally, electrical conductivity, which is crucial for specific applications such as resistance grids, was also investigated.

## 2. Materials and Methods

### 2.1. Alloy Preparation

Eight melts with changing nickel content and chromium addition were prepared using the electrical induction furnace of intermediate frequency IMSK 10 (Inducal Göllingen, Dresden, Germany) with 15 kg capacity crucible (Mammut, Puschwitz, Germany). Charge composition was as follows: sorel metal (Rio Tinto Iron & Titanium, Quebec, QC, Canada), Fe-Mn (Stanchem, Niemce, Poland), technically pure silica (Stanchem, Niemce, Poland), steel scrap (Promel, Radom, Poland, pure nickel (Stanchem, Niemce, Poland) and for castings with chromium addition Fe-Cr (Stanchem, Niemce, Poland). Melts were superheated to 1490 °C each time and kept at this temperature for 2 min. Next, the melts were subjected to spheroidization (Fe-Si-45%-Mg-6%-Ca-1.9%) and inoculation (Fe-Si-75%-Ca-1%-Ba-1%-Al-1%) processes carried out with the bell method. The bell method involves the use of a steel bell attached to a rod with a crucible cover. The bell is cylindrical (3 mm wall thickness, 55 mm inner diameter, 100 mm length) and contains a mixture of inoculant particles (2–5 mm) and nodularizer particles (1–10 mm). The nodularization and inoculation process is performed by immersing the bell into molten metal for approximately 15 s. After these treatments, liquid metal was placed back in the furnace for 2 min. As a last step of the process, melts were poured into molds and for each of the eight compositions, and shafts with a diameter of 25 mm and a length of 220 mm were obtained.

### 2.2. Microstructure Analysis

The polished cross-sections of each casting were examined using a Leica MEF 4M microscope (Leica Microsystems, Wetzlar, Germany) with a QWin v3.5 quantitative analyzer (Leica Microsystems, Wetzlar, Germany) and Keyence VHX-7000 (Keyence Corporation, Osaka, Japan). Also, in addition, a Tescan Mira scanning electron microscope (SEM) (Tescan Orsay Holding, Brno, Czech Republic) equipped with an Oxford Electron Backscatter Diffraction (EBSD) detector (Oxford Instruments, Abingdon, UK) was used for structure analysis.

Thermodynamic calculations of detailed phase diagrams for each casting composition were performed with the Thermo-Calc software (version 2024) equipped with a database for Fe alloys (TCFE14).

### 2.3. Carbides Identification

The detailed carbides analysis, including its identification, was carried out with a probe-corrected Themis (200 kV) transmission electron microscope (TEM) equipped with a field emission gun (FEG) and a windowless four-quadrant Super-X detector (resolution 132 eV) of the integrated Energy dispersive spectroscopy (EDS) system (all by ThermoFisher Scientific, Eindhoven, The Netherlands). For investigations, thin foils were prepared, with the focused ion beam (FIB) method using a Scios dual-beam (ThermoFisher Scientific, Eindhoven, The Netherlands), having the form of rectangular lamellae (~12 μm × ~6 μm × 100 nm). The maps which present the local chemical composition are built of 500 × 500 pixels using ‘net counts’ and were acquired with a 0.5 nm electron probe (Thermo Fisher Scientific, Eindhoven, The Netherlands). A semi-quantitative analysis of the chemical composition of individual phases was carried out by overlaying elemental distribution maps and integrating signal intensities within a 100 nm × 100 nm area. Quantification was carried out using a standardless approach based on a set of Cliff–Lorimer coefficients provided by the manufacturer, without applying absorption or fluorescence corrections. This approach allows to keep measurement error less than 5%.

### 2.4. Thermal Stability

The thermal stability tests were performed in low and high temperatures by X-ray diffraction (XRD), using Cu Kα radiation. For the tests the Empyrean powder diffractometer by Panalytical (Malvern Panalytical, Almelo, The Netherlands), equipped with Oxford Cryosystems PheniX cryocooler (20–300K) (Oxford Cryosystems, Oxford, UK) and Anton Paar HTK 1200N oven (300–1200K) (Anton Paar GmbH, Graz, Austria), were used. Low temperature data were collected in high vacuum (10^−7^ mbar), while the high temperature studies were made in an air atmosphere. For the measurements, the samples were cut into thin plates (3 mm of height). In both cases the sample stage position was calibrated for thermal displacements. The XRD data were evaluated using the Rietveld method.

### 2.5. Electric Conductivity

The measurement of electrical conductivity was performed using the eddy current method. This non-destructive technique is based on the principle of electromagnetic induction, whereby alternating magnetic fields induce circulating eddy currents within electrically conductive materials. The conductivity measurements were conducted using PCE—COM20 (PCE Instruments) device (PCE Instruments, Meschede, Germany). During the measurement process, the instrument converts the complex impedance into a corresponding electrical conductivity value.

## 3. Results and Discussion

### 3.1. Chemical Composition

Table 1 presents the chemical composition of the investigated cast irons. The calculation of the carbon equivalent (CE) was performed based on the following formula [14]:

(1) CE=C%+0.33·Si%+0.047·Ni%−(0.0055·Ni%·Si%)

where the concentrations of C, Si, and Ni are expressed in weight percent (wt.%).

The chemical composition of cast irons 1–4 is based on grade EN GJSA XNi22 [15]; however, some of the castings have nickel content higher than defined. For casting 5–8, the chemical composition is based on EN GJSA XNiSiCr35 5 2 [15] some of prepared samples have nickel content lower than defined. To sum up, for both grades, cast irons with nickel content having approximately 21, 25, 28, and 35 wt.% were prepared.

**Figure 1:**
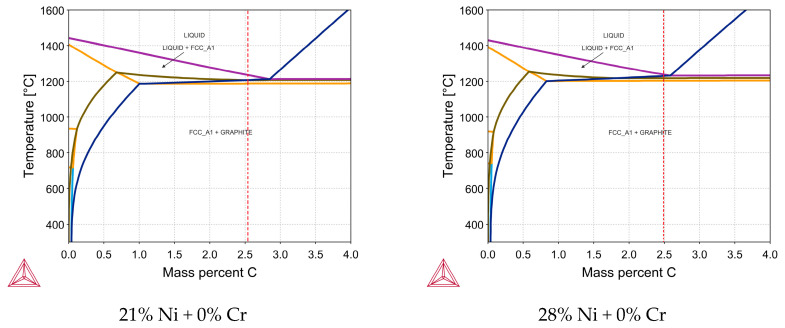

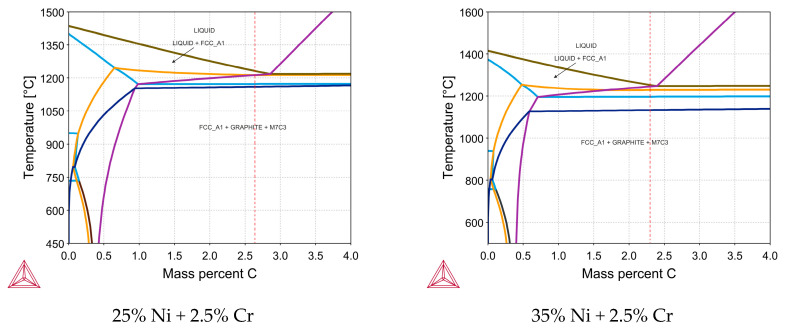
Thermo-Calc results for cast irons No. 1, 3, 6, 8.

Figure 1 presents detailed phase diagrams for exemplary prepared cast irons with main phases crystallized in the investigated system. The red dotted line represents the exact location of the chemical composition in the phase diagram. All prepared castings are slightly hypoeutectic, which is important from the point of view of the comparison of obtained results. As demonstrated in the further part of the work, the phase diagrams calculated using Thermo-Calc software correspond to the crystallization path involving austenite dendrites + graphite eutectic (γ + graphite) in the case of alloys 1–4, and austenite dendrites + graphite eutectic (γ + graphite) + carbide eutectic (γ + M7C3 carbides).

### 3.2. Microstructure and Carbides Identification

**Figure 2:**
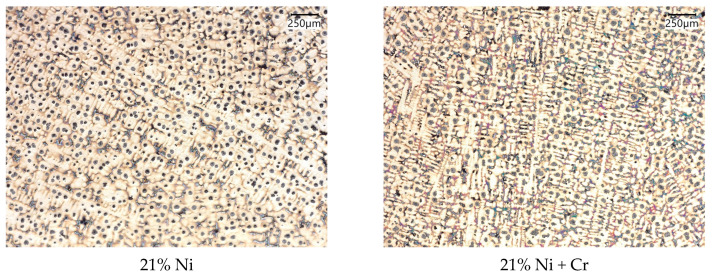

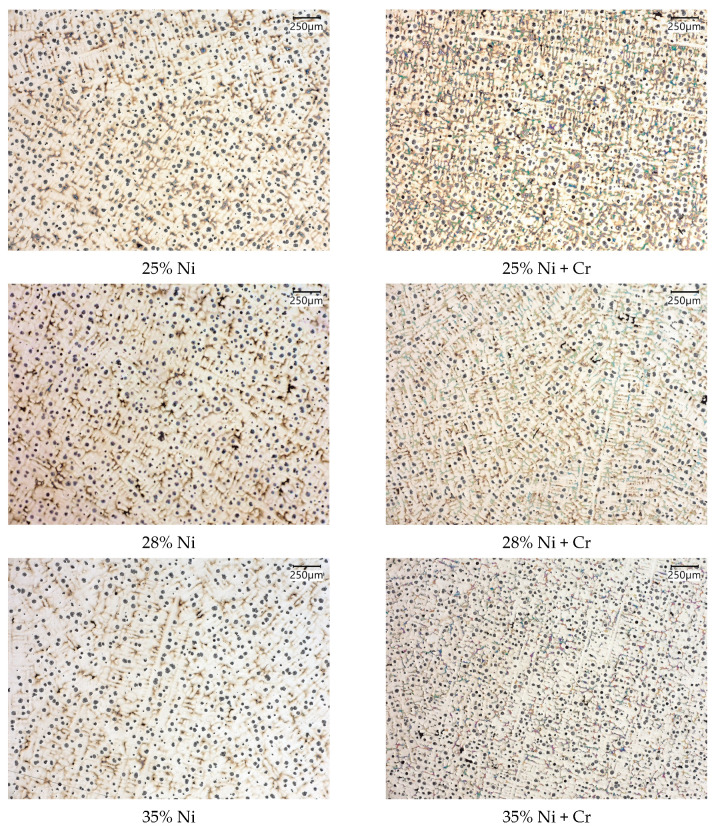
Microstructures of austenitic ductile iron castings, magnification 100×, etched with Nital.

**Figure 3:**
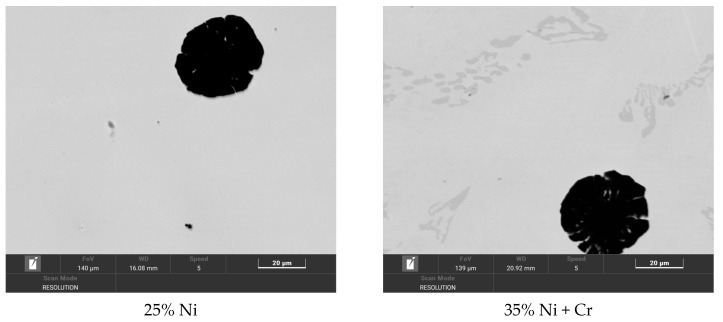
Microstructures of austenitic ductile iron castings, magnification 2000×.

The microstructures at 100 × magnification etched with Nital are shown in Figure 2 and Figure 3. Dendrites of austenite with graphite in the shape of nodules were obtained for all tested cast irons. In addition, eutectic carbides were present in castings 5–8. Despite significant differences in nickel content, in all cases microstructures typical of slightly hypoeutectic compositions were obtained, without the presence of primary graphite, which is consistent with Thermo-Calc predictions, as shown in Figure 1. In general, the good quality of all eight microstructures is observed: graphite distribution is relatively uniform, and shrinkage cavities are not observed. The more detailed analysis of microstructure parameters such as secondary dendrite arm spacing (SDAS), graphite percentage, number of particles per 1 mm^2^ of iron casting surface, degree of spheroidization acc. to ISO 945-1 [16], mean diameter, shape factor, and chromium carbides fraction can be found in our previous works [10,17].

**Figure 4:**
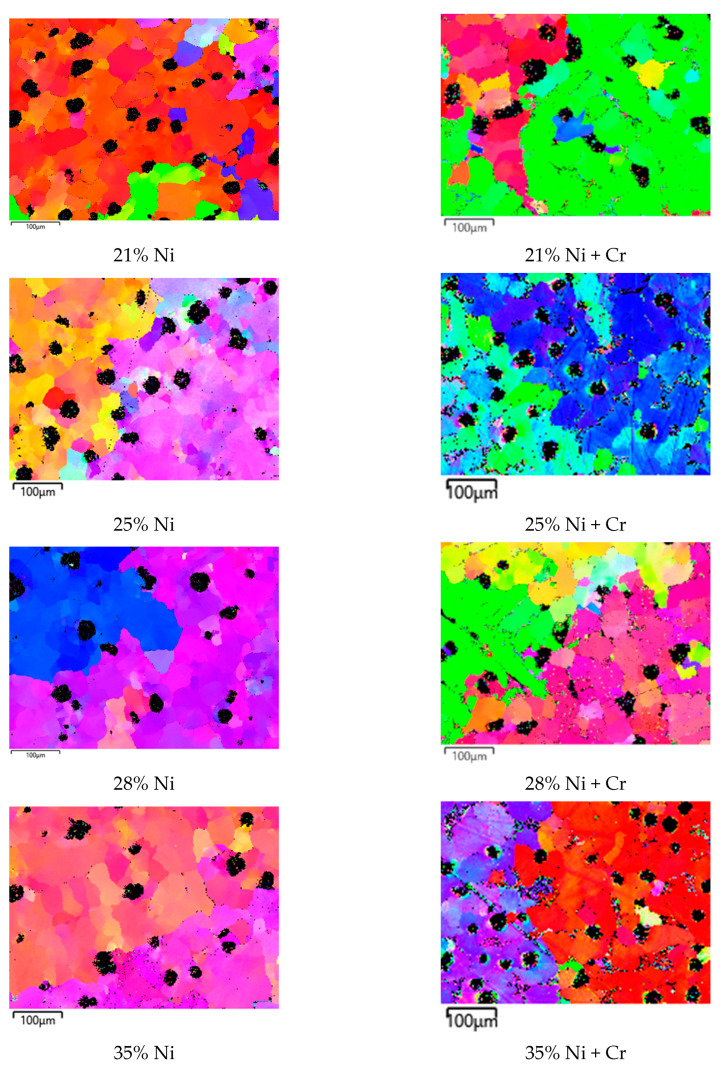
EBSD map for investigated castings, magnification 500×.

The analysis carried out using scanning electron microscopy with an EBSD detector (Figure 4) revealed the presence of graphite nodules’ location in the structures of investigated cast irons. They were observed in interdendritic regions or on the grain boundaries of dendritic grains. It proves that graphite nodules are not absorbed during the crystallization process. This process determines the degree of homogeneity in graphite nodules’ distribution.

The literature review indicated that in investigated cast irons, the carbides should be of type M_7_C_3_ [18,19]. To verify this thesis, TEM analysis was conducted for one of the investigated cast irons, i.e., No. 6 (28% Ni + Cr).

**Figure 5:**
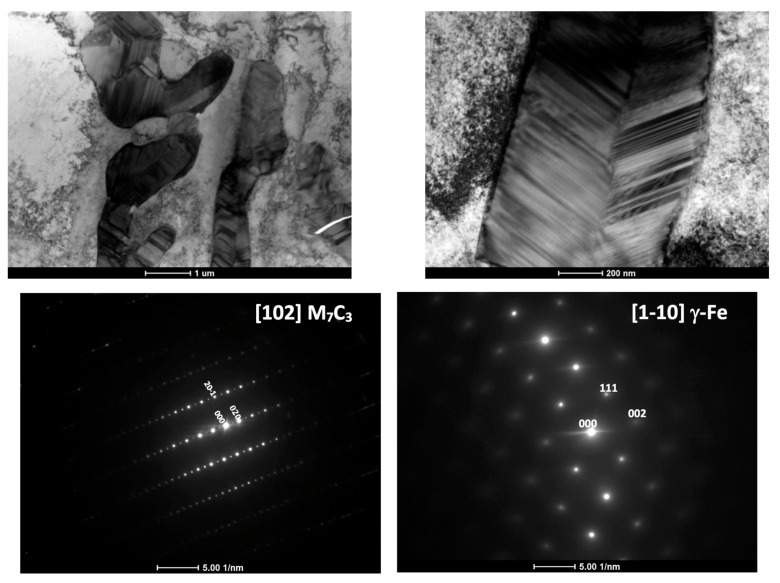
TEM/BF for carbide and matrix from casting No. 6 with 28% Ni + Cr.

TEM/BF observations showed that these carbides contain numerous planar defects, and their outer boundaries were frequently faceted (Figure 5). This morphological feature is characteristic of M_7_C_3_ carbides. The selected area electron diffraction patterns (SAEDP) confirmed that the carbides are of the M_7_C_3_ type and possess a hexagonal structure, which is consistent with the findings of E. Eshed et al. [20], who carried out an in-depth analysis of the atomic structure of M_7_C_3_ carbides. At the interface between the austenitic matrix and the carbides, no decohesion was detected, indicating a good bond between the two phases. No dislocations were observed within the carbide regions.

**Figure 6:**
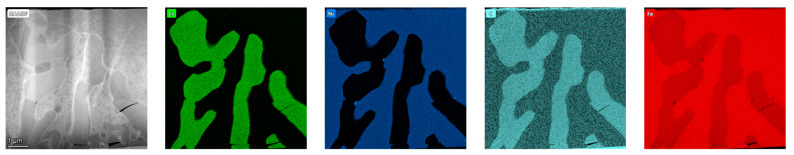
STEM/HAADF image of carbide in casting No. 6; maps presenting distribution of Cr, Ni, C and Fe.

**Figure 7:**
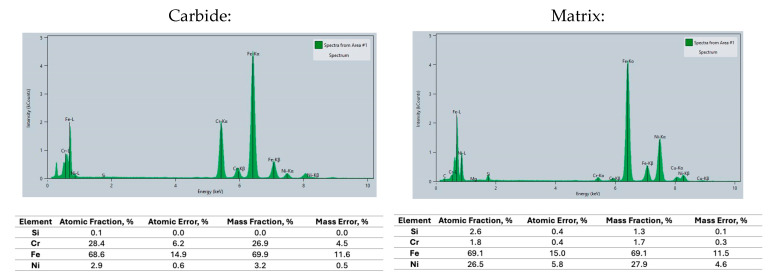
Spectrum for carbide and matrix from casting No. 6 with 28% Ni + Cr.

STEM/EDS maps presenting the distribution of alloying elements within the carbides are presented in Figure 6. They showed that main elements that built the carbide were chromium, carbon and iron. However, the spectrum collected from carbide (Figure 7) shows that a small amount of nickel was also one of its components. Presence of the latter element in M_7_C_3_ at a similarly low level (<3 wt.%) was previously documented in 316L laser synthetized coating [21]. Evidently, it is a threshold concentration of Ni in M_7_C_3_, as anything above it is repulsed by expanding carbide boundary and dissolved in the remaining austenitic matrix. Please note that at certain places where their boundaries met, a local strong enrichment in nickel must have taken place, resulting in the formation of Ni_3_C precipitates [22].

### 3.3. Resistance Towards Oxidation at High Temperatures

**Figure 8:**
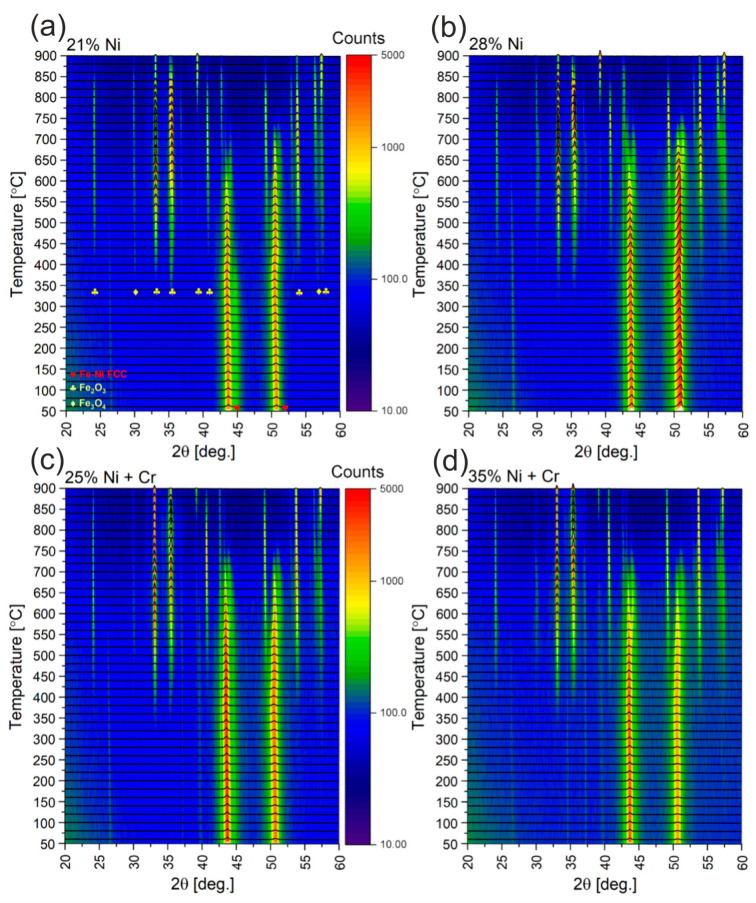
High-temperature XRD patterns recorded for castings No. 1 (**a**), 3 (**b**), 6 (**c**) and 8 (**d**).

For checking thermal stability in air at high temperatures casting, No. 1 (21% Ni), 3 (28% Ni), 6 (25% Ni + Cr) and 8 (35% Ni + Cr) were selected, to cover entire investigated nickel addition—from 21% up to 35%. XRD patterns as a function of the temperature are shown in Figure 8. The samples in the form of solid, thin plates were used for those studies. At room temperature, the only identified phase was Fe-Ni FCC (Face-Centered Cubic), while very weak reflection at about 26.5° of 2θ can be associated with graphite 002 main reflection. For casting with 21% Ni, this phase is visible almost up do 700 °C, while for other castings, the Fe-Ni FCC phase is visible above 700 °C. As the XRD signal is gathered from less than 0.1 mm of the sample depth, the diminishing of the FCC reflections is related to oxidation of the surface. Therefore, the temperatures, at which reflections from the FCC phase disappear, reflect resistance of the alloy towards oxidation. When the temperature rises above 350 °C, the reflections from Fe_2_O_3_ and Fe_3_O_4_ are observed. Initially, the reflections from oxides are relatively small, which suggests the forming of the passivation layer onto the surface of the plate-like samples. At higher temperatures the reflections from the FCC phase promptly disappear, which is likely associated with rapid deterioration of the sample surface.

### 3.4. Thermal Stability at Cryogenic Temperatures

**Figure 9:**
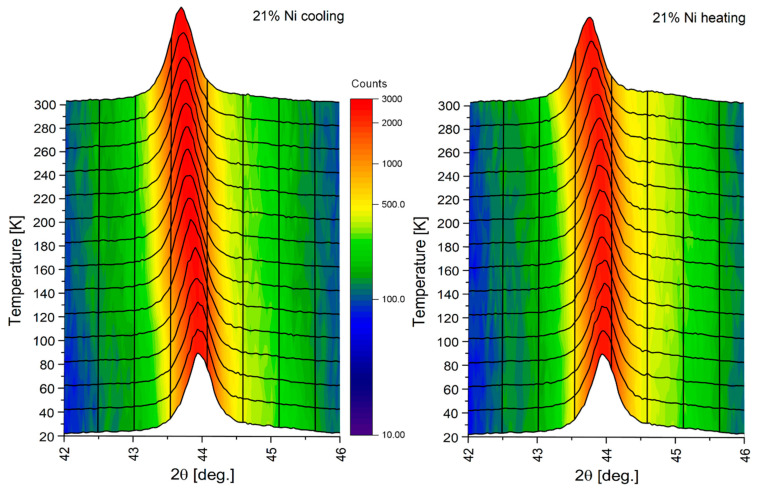
XRD pattern showing temperature evolution of the main 111 reflections originating from the FCC phase for casting No. 1 (21% Ni) upon cooling and heating.

In order to investigate cryogenic temperature behavior, which is essential for the usage of the castings in extreme temperature conditions, XRD measurements were performed upon cooling and heating to track possible irreversibility of the lattice parameters. Figure 9 presents an example of XRD pattern for investigated castings in the enlargement of the area of the main 111 reflections originating from the FCC structure. As the sample position was automatically calibrated for each temperature, the shifts in the reflection are entirely due to the change in the FCC lattice parameter. For some castings, a clear irreversibility was noticed, as depicted in Figure 9. As apparent, the position of the reflection changes in a different manner during cooling and heating, suggesting hysteresis of the lattice parameter, when casting is exposed to low temperatures and heated again to the room temperature.

**Figure 10:**
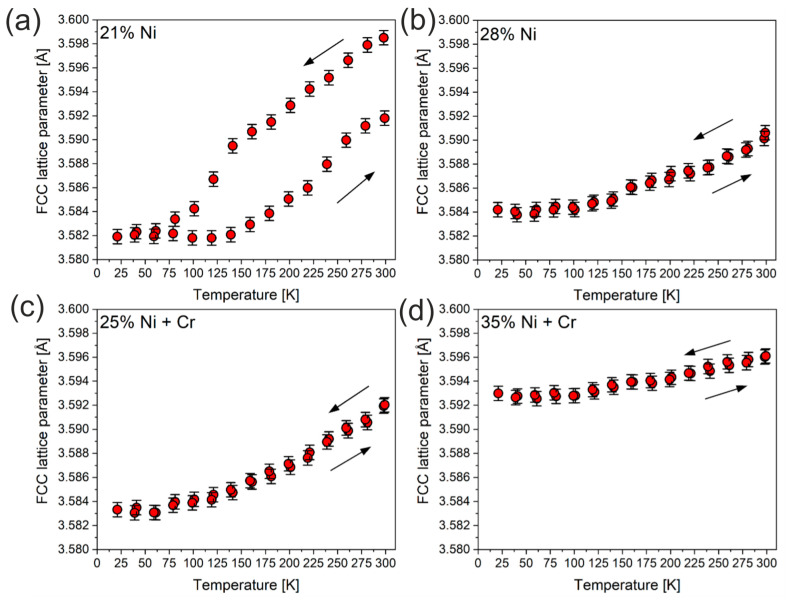
Lattice parameters for castings No. 1 (**a**), 3 (**b**), 6 (**c**) and 8 (**d**) versus temperature at cryogenic conditions. The scales are the same for all panels to facilitate tracking differences.

To quantify this behavior, the patterns were analyzed in terms of the Rietveld refinements. The resulting lattice parameters versus temperature (20–300K) are presented in Figure 10. Typically for simple metals/alloys, one could expect expansion of the lattice parameter during heating, which was indeed observed. Nonetheless, the FCC lattice parameter of casting with 21% Ni at 300 K does not have the same value after the cooling down and heating process, clearly showing hysteresis. For other castings with 25, 28 and 35% of Ni, the irreversibility was not evidenced. Interestingly, as the Ni content in the casting rises, the temperature dependence of the lattice parameter flattens. This is clearly observed for 35% Ni (Figure 10d), where the thermal expansion is significantly lower than for castings with smaller Ni concentration. The changes in lattice dynamics are solely responsible for this behavior. Namely, the anharmonicity of the FCC lattice is significantly reduced, mainly due to restricting the maximum atom displacements from the equilibrium positions. Therefore, the harmonic approximation of the Morse potential becomes valid at higher temperatures. The hysteresis visible for casting with 21% Ni can be attributed to the well-known austenitic–martensitic transformation that due to plastic strains can occur at cryogenic temperatures [23]. The martensite precipitations of about 20% from the austenite phase were confirmed by SEM and XRD studies of the 21% Ni sample after the cryogenic cycle.

### 3.5. Electrical Conductivity

The results of the measured electrical conductivity are summarized in Table 2. Measured values indicate that the electrical conductivity of investigated austenitic spheroidal cast iron is at a low level, especially when it is compared to materials known as good conductors, such as copper with conductivity 58 MS/m at room temperature [24]. On the other hand, the measured values are similar to superalloy Inconel 100—1.3–1.5 MS/m, having ~60 wt.% Ni and 10 wt.% Cr [24].

The electrical conductivity at the presented level might be explained by a few factors. First is the presence of austenite in the matrix, which has lower electrical conductivity (1.3–1.5 MS/m [25]) than ferrite (~10 MS/m [25]) or pearlite (3–7 MS/m [25]), and nickel is responsible for austenite stabilization. The second reason is the presence of carbides in castings with chromium addition, which interrupt the continuity of the crystal lattice. As a consequence, casting with the same nickel content, but with the addition of chromium has lower electrical conductivity than casting without chromium addition.

## 4. Conclusions

Based on this research, the following conclusions can be drawn:

1. In investigated slightly hypo-eutectic ductile iron with a nickel range of 21–35 wt.%, the highly branched dendritic microstructure is predominantly present with graphite nodules located in the interdendritic regions and on the austenite grain boundaries. The highly branched austenitic dendrites determine the uniform distribution of graphite nodules which results in a high-quality engineering material.
2. TEM microanalysis revealed that austenitic metallic matrix in chromium-alloyed ductile iron contains Cr = 1.7 ± 0.3 wt.% next to eutectic carbide M_7_C_3_ type.
3. XRD analysis shows the high thermal stability of high-nickel ductile iron. In particular, it has been proved that the nickel content plays a key role in thermal stability under cryogenic conditions. The minimum required nickel content of 25 wt.% was satisfactory to provide thermal stability at cryogenic temperatures down to 25 K.
4. The electrical conductivity characteristic for investigated austenitic high-nickel cast iron was provided, and the attained values are at the level of Ni-based superalloys.

## Figures and Tables

**Table 1:** The summary of the chemical composition of the experimental cast irons [10].

Cast Iron	Composition [%]								CE%
	C	Cr	Ni	Si	Mn	Mg	S	Fe	
1	2.74	0.00	21.14	2.27	1.99	0.05	0.02	Balance	4.22
2	2.82	0.00	25.03	2.00	2.05	0.05	0.02	Balance	4.38
3	2.50	0.00	28.04	2.11	1.90	0.05	0.02	Balance	4.19
4	2.47	0.00	34.32	2.00	2.02	0.05	0.01	Balance	4.37
5	2.71	2.70	21.25	2.00	1.01	0.05	0.01	Balance	4.14
6	2.64	2.53	24.67	1.94	0.95	0.05	0.02	Balance	4.18
7	2.82	2.67	28.67	2.03	1.02	0.05	0.01	Balance	4.52
8	2.30	2.46	35.80	1.87	1.01	0.05	0.02	Balance	4.23

**Table 2:** Electrical conductance measured for all castings.

Electrical Conductivity, MS/m							
21% Ni		25% Ni		28% Ni		35% Ni	
0% Cr	2.5% Cr	0% Cr	2.5% Cr	0% Cr	2.5% Cr	0% Cr	2.5% Cr
1.69	1.63	1.41	1.33	<0.5	<0.5	<0.5	<0.5

## Data Availability

The original contributions presented in this study are included in the article. Further inquiries can be directed to the corresponding author.

## References

[1] American Foundrymen’s Society (1993). Ductile Iron Handbook, Des Plaines.

[2] Yang Y.L., Cao Z.Y., Lian Z.S., Yu H.X. (2011). A study on microstructure of ductile Ni-resist cast iron for exhaust manifolds and mechanical property at the condition of alternative thermal cycles. Adv. Mater. Res..

[3] Janus A., Granat K. (2014). Heat treatment of Ni–Mn–Cu cast iron. Arch. Civ. Mech. Eng..

[4] Kühn H.-J. (2017). Thermomechanical fatigue of heat-resistant austenitic cast iron EN-GJSA-XNiSiCr35-5-2 (Ni-Resist D-5S). Int. J. Fatigue.

[5] Alabbasian F., Boutorabi S.M.A., Kheirandish S. (2016). Effect of inoculation and casting modulus on the microstructure and mechanical properties of ductile Ni-resist cast iron. Mater. Sci. Eng. A.

[6] Moe G. (2022). Properties and Applications of Ni-Resist and Ductile Ni-Resist Alloys.

[7] Fragassa C., Radović N., Pavlović A., Minak G. (2016). Comparison of mechanical properties in compacted and spheroidal graphite irons. Tribol. Ind..

[8] Rashidi M.M., Hasbullah I.M. (2014). The effects of solidification on the microstructure and mechanical properties of modified ductile Ni-resist iron with a high manganese content. Mater. Sci. Eng. A.

[9] Rashidi M.M., Idris M.H. (2013). Microstructure and mechanical properties of modified ductile Ni-resist with higher manganese content. Mater. Sci. Eng. A.

[10] Bork M., Chulist R., Górny M., Kowalczyk M., Marosz J. (2025). The influence of nickel content on the structure parameters and magnetic properties of austenitic ductile iron castings. Arch. Civ. Mech. Eng..

[11] Yaakob K.I., Maarof M.R., Rosnizan M.A., Zalani M.R., Ahmad A.H. (2023). Evaluation of dendritic structure of modified ductile Ni-resist. J. Adv. Res. Appl. Mech..

[12] Yaakob K.I., Maarof M.R., Rosnizan M.A., Zalani M.R., Ahmad A.H. (2023). Evaluation of graphite size of modified ductile Ni-resist with higher manganese. J. Adv. Res. Appl. Mech..

[13] Rosnizan M.A., Zalani M.R., Yaakob K.I., Syairah F., Maarof M.R. (2023). Evaluation of modified ductile Ni-resist alloy solidification with higher chromium and manganese. J. Adv. Res. Appl. Mech..

[14] Fatahalla N., AbuElEzz A., Semeida M. (2009). C, Si and Ni as alloying elements to vary carbon equivalent of austenitic ductile cast iron: Microstructure and mechanical properties. Mater. Sci. Eng. A.

[15] (2002). Founding—Austenitic Cast Irons.

[16] (2019). Microstructure of Cast Irons—Part 1: Graphite Classification by Visual Analysis.

[17] Bork M., Górny M., Palumbo G., Angella G., Marosz J. (2025). Microstructure, mechanical parameters, and corrosion resistance of austenitic ductile iron castings. Arch. Civ. Mech. Eng..

[18] Ma S., Xing J., He Y., Li Y., Huang Z., Liu G., Geng Q. (2015). Microstructure and crystallography of M7C3 carbide in chromium cast iron. Mater. Chem. Phys..

[19] Wiengmoon A., Chairuangsri T., Brown A., Brydson R., Edmonds D.V., Pearce J.T.H. (2005). Microstructural and crystallographical study of carbides in 30 wt% Cr cast irons. Acta Mater..

[20] Eshed E., Choudhuri D., Osovski S. (2022). M7C3: The story of a misunderstood carbide. Acta Mater..

[21] Gorunov A.I. (2020). Investigation of M7C3, M23C6 and M3C carbides synthesized on austenitic stainless steel and carbon fibers using laser metal deposition. Surf. Coat. Technol..

[22] He L. (2010). Hexagonal close-packed nickel or Ni3C?. J. Magn. Magn. Mater..

[23] Ryś M. (2015). Modeling of Damage Evolution and Martensitic Transformation in Austenitic Steel at Cryogenic Temperature. Arch. Mech. Eng..

[24] Warlimont H., Martienssen W. (2018). Springer Handbook of Materials Data.

[25] (2023). Granta Selector 2023 R1Version.

